# Low Molecular Weight Heparin Induced Skin Necrosis without Platelet Fall Revealing Immunoallergic Heparin Induced Thrombocytopenia

**DOI:** 10.1155/2013/849168

**Published:** 2013-11-03

**Authors:** Thomas Godet, Sébastien Perbet, Aurélien Lebreton, Guillaume Gayraud, Sophie Cayot, Aymeric Tremblay, Aurélie Ravinet, Sébastien Christophe, Renaud Guérin, Julien Pascal, Matthieu Jabaudon, Amr Hassan, Anne-Françoise Sapin, Jean-Etienne Bazin, Jean-Michel Constantin

**Affiliations:** ^1^CHU Clermont-Ferrand, CHU Estaing, Réanimation Adultes & Unité de Soins Continus, 1 Place Lucie et Raymond Aubrac, 63003 Clermont-Ferrand Cedex 1, France; ^2^Faculté de Médecine, Université d'Auvergne, R2D2, EA 7281, INSERM, Place Henri-Dunant, 63000 Clermont-Ferrand, France; ^3^Faculté de Médecine, Université d'Auvergne, Centre de Simulation CAUMSSI, Place Henri-Dunant, 63000 Clermont-Ferrand, France; ^4^CHU Clermont-Ferrand, CHU Estaing, Hematology, 1 Place Lucie et Raymond Aubrac, 63003 Clermont-Ferrand Cedex 1, France; ^5^CHU Clermont-Ferrand, CHU Estaing, Anatomopathology, 1 Place Lucie et Raymond Aubrac, 63003 Clermont-Ferrand Cedex 1, France

## Abstract

Low molecular weight heparins (LMWH) are commonly used in the ICU setting for thromboprophylaxis as well as curative decoagulation as required during renal replacement therapy (RRT). A rare adverse event revealing immunoallergic LMWH induced thrombopenia (HIT) is skin necrosis at injection sites. We report the case of a patient presenting with skin necrosis witnessing an HIT after RRT, without thrombocytopenia. The mechanism remains unclear. Anti-PF4/heparin antibodies, functional tests (HIPA and/or SRA), and skin biopsy are of great help to evaluate differential diagnosis with a low pretest probability 4T's score.

## 1. Introduction

Renal failure is a frequent condition observed among ICU patients. Renal replacement therapy (RRT) is often required to treat this affection. Curative anticoagulation with heparin derivatives is mandatory to prevent filter clotting especially during proaggregant conditions such as sepsis. We wish to report a rare complication of heparin treatment with enoxaparin with a low pretest probability 4T's score [1], due to an immunoallergic heparin induced thrombopenia (HIT): skin necrosis at injection site. 

## 2. Case Report

A 57-year-old woman with a history of severe melancholic state was hospitalized in psychiatric ward for 4 months before admission to our hospital. She presented in the emergency department with arterial hypotension and hypothermia due to severe dehydration because of severe feeding troubles with self-induced vomiting, anorexia, and loss of weight. The patient had no history of previous exposition to heparin derivatives. Platelet count was 490 G/L. 

Biological investigations found severe acute kidney injury (creatinine 720 *μ*mol/L and urea 37 mmol/L), metabolic acidosis (pH 7.10, HCO3–14 mmol/L), and mild hyperkaliemia of 5.4 mmol/L. Abdominal scanning found no renal or digestive abnormality. First line treatment consisted of important fluid loading with crystalloids (5000 mL ringer lactate, 500 mL sodium bicarbonate 1,4%, and 500 mL sodium bicarbonate 4,2%) and parallel norepinephrine infusion to obtain satisfying hemodynamics. The patient was then transferred to our ICU. 

The hospitalization course was marked with an acute respiratory distress syndrome due to acute aspiration, cardiac failure with infusion of inotropic agent, and persistent anuric renal failure with the need of RRT (continuous venovenous hemofiltration (CVVHF), using the Aquarius System, Edwards Lifesciences, France, blood flow 200–250 mL/min, and ultrafiltrate flow 35 mL/kg/h, priming with 5000 UI unfractionated heparin (UFH) in 2000 mL saline solution and Hemosol substitution solution delivered one-third prefilter and two-thirds postfilter). 

**Figure 1:**
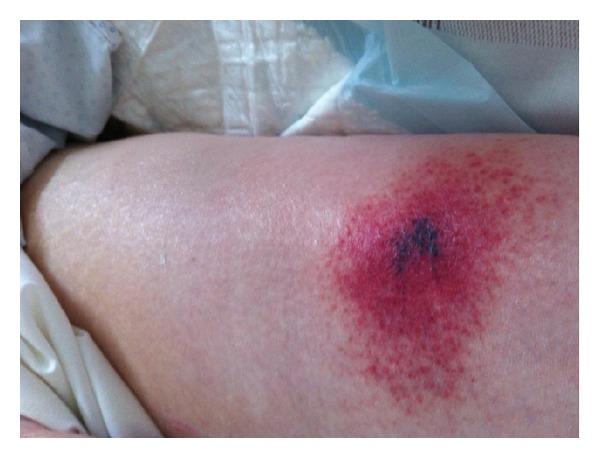
Skin necrosis lesion at injection sites of left thigh.

**Figure 2:**
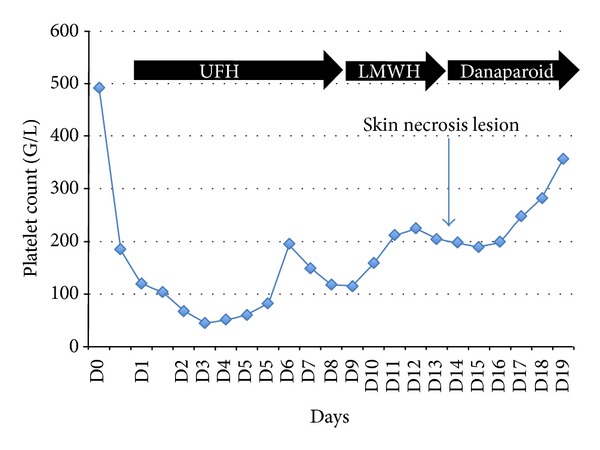
Timeline of platelets count and anticoagulant therapies. Skin necrosis appeared on day 15. Platelet count increased after danaparoid initiation. UFH: unfractionated heparin; LMWH: low molecular weight heparin; RRT: renal replacement therapy.

**Figure 3:**
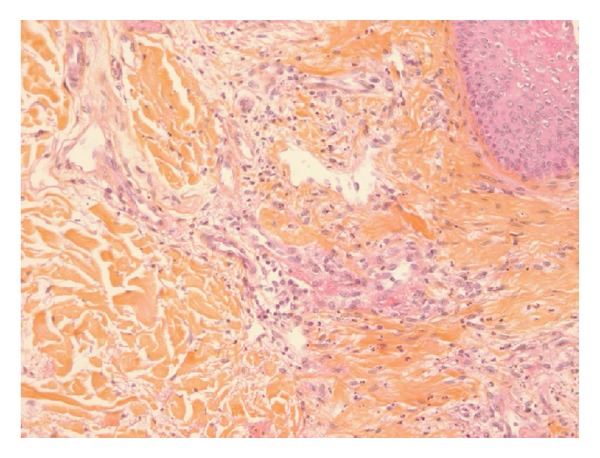
Skin biopsy showing leukocytoclastic vasculitis and intravascular microthrombi.

Anticoagulation was started with UFH during RRT until day 9. Thromboprophylaxis was started on day ten with low molecular weight heparin (LMWH) and enoxaparin sodium and (40 UI s.c./24 h). On day fifteen, the diagnosis of immunoallergic HIT was suspected following the observation of painful erythematous lesions at the injection sites of LMWH and rapid evolution to skin necrosis (Figure 1). Bilateral iliac veins thrombosis was found on ultrasound examination (femoral central venous access). No RRT filter clotting event occurred. A switch to danaparoid sodium (Orgaran, Organon, France) anticoagulation was started and immunoassay for immunoallergic HIT (GTI PF4 Enhanced, GTI Diagnostics, Waukesha, WI) revealed the presence of IgG class of anti-PF4/heparin antibodies (1,36 OD). The heparin induced platelet activation (HIPA) assay was negative and the serotonin release assay (SRA) was positive. Platelet count was normal (198 G/L, Figure 2). Necrotic lesions disappeared within five days after start of alternative anticoagulation with danaparoid sodium. Notably, our patient presented with a 4T's score of 3 in ICU. Skin biopsy found leukocytoclastic vasculitis and thrombi (Figure 3).

## 3. Discussion

Heparin induced skin lesions at injection site (or elsewhere) are not strongly associated with immunoallergic HIT but most likely due to delayed-type hypersensitivity (DTH) [2, 3]. The well-established scoring system developed by Lo et al. [4] has recently been modified, downgrading nonnecrotizing skin lesions from 1 to 0 point in the fourth T (oTher) [1]. Necrotic skin lesions are manifestations of immunoallergic HIT and are scored with 2 points. Skin biopsies frequently show thrombosis in the dermal microvascular and biological tests (IgG class of anti-PF4/heparin antibodies, HIPA, and SRA) which are in favor of immune activation of platelets. 

Necrotizing skin lesions have been described more frequently with UFH treatments [5, 6]. The occurrence of such skin lesion has been reported in a small number of cases (about 30 reports in MedLine in August 2013) and a recent prospective study found no incidence over a population of 87 patients with skin lesions due to LMWH subcutaneous injections [2]. In a previous publication, Warkentin reported an incidence of 10 to 20% among patients with positive anti-PF4/heparin antibodies [7]. 

These two types of skin lesions are due to intradermal microvascular thrombosis, possibly explained by the presence of Fc*γ*RIIa receptors on the endothelial cells of the superficial dermal vascular plexus [8], which are similar to those on platelets and bound by the ternary complexes of antibody-heparin-platelet factor 4. 

The paradoxal absence of thrombocytopenia in patients diagnosed with HIT is especially observed in the ICU setting [9]. Several acute conditions are accompanied with thrombocytosis (sepsis, inflammation, trauma, drug secondary effects, etc.) [10]. This situation reflects a balance between production and consummation of platelets and can mask thrombocytopenia due to intravascular HIPA, as previously reported [11].

The similar clinical presentations—at least in the first days—of necrotizing and nonnecrotizing lesions might lead to confusion and mis-detection of HIT diagnosis, with the risk of life-threatening thromboembolic complications if heparin treatment is not substituted. In order to avoid such defect, clinicians should be aware of the existence of such skin lesion and have routine inspection of injection sites of heparin derivatives. In case of suspected cutaneous reaction, the use of the 4T's scoring system is recommended and guides further biological investigations. Skin biopsy is a key exam for differential diagnosis, possibly ruling out HIT if no vessel occlusion is detected [12].

Interestingly, a recent report found a higher incidence of skin necrosis due to HIT with the use of the recently Food and Drug Administration approved generic enoxaparin, raising the possibility of defects in purity of enoxaparin molecules [13].

## 4. Conclusion

Heparin induced skin necrosis at injection site is an extremely rare complication of administration of LMWH. The pathomechanism remains uncertain but involves intravascular platelet activation and consequent formation of microvasculature clotting in poorly vascularized dermal layer. Anti-PF4/heparin antibodies, functional tests (HIPA and/or SRA), and skin biopsy are of great help to evaluate differential diagnosis with a low pretest probability 4T's score.
